# Cellular Localization and Associations of the Major Lipolytic Proteins in Human Skeletal Muscle at Rest and during Exercise

**DOI:** 10.1371/journal.pone.0103062

**Published:** 2014-07-23

**Authors:** Rachael R. Mason, Ruth C. R. Meex, Aaron P. Russell, Benedict J. Canny, Matthew J. Watt

**Affiliations:** 1 Biology of Lipid Metabolism Laboratory, Department of Physiology, Monash University, Clayton, Victoria, Australia; 2 Centre of Physical Activity and Nutrition (C-PAN) Research, School of Exercise and Nutrition Sciences, Deakin University, Burwood, Victoria, Australia; INSERM/UMR 1048, France

## Abstract

Lipolysis involves the sequential breakdown of fatty acids from triacylglycerol and is increased during energy stress such as exercise. Adipose triglyceride lipase (ATGL) is a key regulator of skeletal muscle lipolysis and perilipin (PLIN) 5 is postulated to be an important regulator of ATGL action of muscle lipolysis. Hence, we hypothesized that non-genomic regulation such as cellular localization and the interaction of these key proteins modulate muscle lipolysis during exercise. PLIN5, ATGL and CGI-58 were highly (>60%) colocated with Oil Red O (ORO) stained lipid droplets. PLIN5 was significantly colocated with ATGL, mitochondria and CGI-58, indicating a close association between the key lipolytic effectors in resting skeletal muscle. The colocation of the lipolytic proteins, their independent association with ORO and the PLIN5/ORO colocation were not altered after 60 min of moderate intensity exercise. Further experiments in cultured human myocytes showed that PLIN5 colocation with ORO or mitochondria is unaffected by pharmacological activation of lipolytic pathways. Together, these data suggest that the major lipolytic proteins are highly expressed at the lipid droplet and colocate in resting skeletal muscle, that their localization and interactions appear to remain unchanged during prolonged exercise, and, accordingly, that other post-translational mechanisms are likely regulators of skeletal muscle lipolysis.

## Introduction

Lipolysis is a highly conserved function that involves the sequential breakdown of triacylglycerol (TAG) to produce free fatty acids that are mostly used for energy production. Much of our understanding of lipolysis is derived from studies of adipose tissue metabolism. Lipolysis is regulated by a complex interplay involving the phosphorylation, trafficking and interaction of several key proteins including, perilipin 1 (PLIN1) and adipose triglyceride lipase (ATGL). Perilipin 1 is a critical modulator of adipocyte TAG lipolysis by orchestrating protein-protein interactions at the surface of lipid droplets, which contain TAG. During spontaneous (basal) lipolysis some ATGL resides on the lipid droplet but its lipase activity is relatively low because its activator protein, comparative gene identification 58 (CGI-58) [1], associates with PLIN1 [2]. During β-adrenergic (stimulated) lipolysis, protein kinase A (PKA) phosphorylates PLIN1 resulting in its dissociation from CGI-58 [3]. As a consequence, CGI-58 is able to bind and activate ATGL. The outcome of these reactions is a shift from storage to mobilization of fatty acids from triacylglycerol [4], [5]. PKA also promotes the rapid translocation of hormone sensitive lipase (HSL) from the cytosol to the lipid droplet, which interacts with PLIN1 and contributes to maximal lipolysis [6]. However, PLIN1 expression is restricted to adipocytes and steroidogenic tissues [7], raising the possibility that other proteins perform similar functions to PLIN1. Alternatively, lipolysis may be regulated in a cell autonomous manner and PLIN1 is not required for lipolysis in other metabolically active tissues, such as skeletal muscle.

Four proteins with protein sequence homology to PLIN1 were identified and have recently been denoted PLIN2-5 [8]. PLIN proteins are characterized as having common N-terminal motifs, and/or an 11-mer repeat sequence that is predicted to fold into amphipathic helices. However, they differ from one another with respect to mass, cellular localization, transcriptional regulation and protein structure, indicating the likelihood of diverse cellular functions. Although the importance for each PLIN family member is being established, PLIN5 appears to be a major modulator of skeletal muscle lipid metabolism. PLIN5 is expressed in highly oxidative tissues such as red skeletal muscle and heart, and in liver during fasting [9]–[11]. Cell studies show that PLIN5 is localized throughout the cytosol and moves to the surface of the lipid droplet with fatty acid loading [10], [12], where it appears to transport lipids to larger lipid droplets for longer-term storage [13]. Mitochondrial localization of PLIN5 has also been reported, suggesting a role in fatty acid oxidation [14]. Thus, the exchangeable lipid droplet binding properties for PLIN5 indicates that this protein may be involved in the acute regulation of lipid storage / utilization, such as during periods of nutrient deprivation (e.g. fasting/starvation) or a physiological stress such as exercise. As mentioned, the regulation of TAG lipolysis is dependent on the subcellular targeting and trafficking of specific proteins [15]. PLIN5 interactions with ATGL, CGI-58 and HSL were shown in immortalized cell lines overexpressing recombinant proteins [16], [17]. This colocalization is not apparent for other PLIN proteins, supporting the premise of functional specificity for PLIN5. Thus, PLIN5 coordinates the interaction of lipolytic proteins, which may be critical for regulating tissue lipid levels. Indeed, PLIN5 null mice store less TAG and fatty acid in the heart and skeletal muscle compared with wild type mice [18].

PLIN5 associates with lipid droplets and this is not altered in isolated rat skeletal muscle with acute contraction [19] and studies using immunoprecipitation approaches indicate that PLIN5 may control the association of ATGL and CGI-58 to regulate contraction-induced lipolysis [20]. The interaction between PLIN5, ATGL and CGI-58 in human skeletal muscle is currently unknown. The aims of the present study were to examine the cellular localization of PLIN5 and the colocation of other major lipolytic modulators in skeletal muscle at rest and during acute moderate-intensity exercise.

## Methods

### Ethical approval

All subjects participated in the study after being informed of the procedures and associated risks and written consent was obtained in accordance with the Declaration of Helsinki [21], and was approved of by the Monash University Human Research Ethics Committee (CF09/3091 – 2009001685).

### Human Study

#### Subjects and experimental design

Nine recreationally active male subjects (23±1 years, 77±2 kg) participated in a previously published study [22]. Muscle samples from seven of these nine subjects were available for immunohistochemical analysis in this study. Subjects visited the laboratory on three occasions. On the first occasion, peak pulmonary oxygen uptake (VO_2_ peak) was determined during an incremental cycling test to volitional exhaustion (Lode, Groningen, The Netherlands). Expired O_2_ and CO_2_ were collected and analyzed on-line and ventilation determined (AEI Technologies, Pittsburgh, PA). At least three days later, subjects performed a practice trial, which consisted of 60 min cycling at a workload corresponding to 60% VO_2_ peak. VO_2_ was obtained to confirm the subjects' workload. On the day of testing, subjects arrived following an overnight fast. Subjects rested in a supine position and a Teflon catheter was inserted into an antecubital vein. A blood sample was drawn and the line was kept patent by intermittent injection of heparinized saline. A resting muscle sample was obtained from a percutaneous needle biopsy of the *vastus lateralis* with suction, previously prepared with a small incision through the skin and deep fascia under local anesthesia (1% Lidocaine, no adrenaline). The muscle sample was coated in OCT Tissue-Tek (Sukara, Finetek, The Netherlands) and frozen in melting isopropanol frozen in liquid nitrogen before being placed in liquid nitrogen, then stored at −80°C. The subject commenced cycling on the cycle ergometer for 60 min at 60% VO_2_ peak. Expired gases were obtained for 3 min at 15 min intervals. Venous blood and muscle samples were obtained immediately before and after 5 and 60 min of cycling exercise. Subjects were permitted to drink water *ad libitum* during the exercise bout.

### Immunohistochemistry

**Figure 1:**
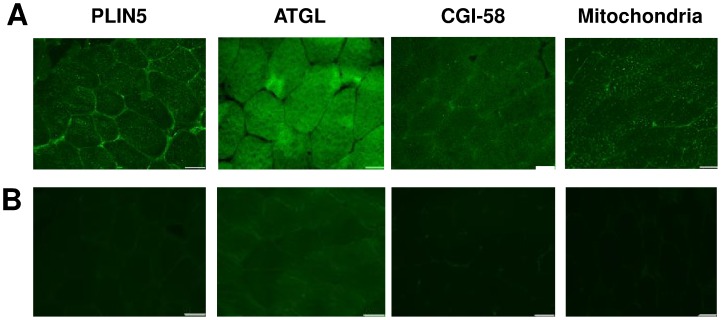
Specificity of PLIN5, ATGL, CGI-58 and Mitochondria immunohistochemistry. (A) Representative images of muscle sections stained with primary antibodies to PLIN5, ATGL, CGI-58 and Mitochondria (OXPHOS). (B) Concentration-matched isotype negative controls, including guinea pig serum, mouse IgG_2a_, rabbit IgG and pre-absorbed OXPHOS with mouse IgG_1_ and IgG_2a_ respectively (Scale bar  = 50 µm).

Serial 10 µm sections were cut at −20°C on to SuperFrost Ultra Plus glass slides. Slides were fixed in Bouin's solution with 0.1% Triton X-100 (Sigma-Aldrich) for 1 h then rinsed 3×5 min in PBS with 0.5% BSA. Sections were blocked in PBS with 1% BSA for 1 h then incubated overnight with the following antibodies: PLIN5 (Cat. no. GP31, Progen Biotechnik, Germany, ∼0.02 mg/ml); ATGL (Cat. no. 2138, Cell Signaling, MA, USA or Cat. no. NBP1-25852, Novus Biologicals, CO, USA, ∼0.02 mg/ml.); CGI-58 (Cat. no. ab73551, Abcam, Cambridge, UK, ∼0.05 mg/ml). Mitochondrial staining was performed using the mitochondrial antibody (Total OXPHOS Cat. no. ab110413, Abcam, Cambridge, UK, ∼0.03 mg/ml), which is directed against the oxidative phosphorylation complexes I–V. For negative controls, primary antibodies were substituted for concentration matched guinea pig serum for PLIN5 (Antibodies Australia), mouse IgG_2a_ for ATGL (Dako, Denmark), rabbit IgG_1_ for CGI-58 (Dako, Denmark) antibodies, or OXPHOS cocktail pre-absorbed 4∶1 overnight with mouse IgG_1_ and IgG_2a_ (Dako). Following a further 3×5 min PBS with 0.5% BSA wash, the corresponding secondary Alexa Fluor antibodies were added (Life Technologies, NY, USA). Representative images of selected polyclonal antibodies (PLIN5, ATGL, CGI-58 and OXPHOS) with isotype matched negative controls shown in Figure 1. Single label slides were examined to ensure minimal bleed-through before dual staining images were captured. Following a 5 min wash in PBS with 0.5% BSA, sections were stained with freshly made, filtered oil red O (Sigma-Aldrich) made in 60% triethylphosphate (Sigma-Aldrich). Slides were washed for 10 min under running tap water then covered with a coverslip. Images were captured using a Zeiss microscope (Zeiss, Oberkochen, Germany) with an AxioCam MR camera using DAPI UV (340–380 nm), FITC (465–495 nm) and Red (568 nm) excitation filters. An average of 20 fibers per subject, per time point was analyzed (5 fibers per section in 4 sections).

### Intramyocellular neutral lipid content

Intramyocellular lipid content was assessed according to the methods of van Loon et al. [23]. Briefly, slides were fixed in Bouin's solution with 0.1% Trition X-100 (Sigma-Aldrich) for 30 min. Sections were stained for lipid droplets with freshly made, filtered oil red O (Sigma-Aldrich) made in 60% triethylphosphate (Sigma-Aldrich).

### Cell culture

Human primary myoblasts were grown in low glucose DMEM, 1% 50 IU/ml penicillin/5 µg/ml streptomycin (P/S), 20% fetal bovine serum, 0.01% bovine fetal growth factor on an extracellular collagen matrix coated coverslip. Once confluent, myoblasts were differentiated for 5 days in media containing low glucose DMEM with 1% P/S and 2% horse serum (Gibco, GK1W2013). Once differentiated to myotubes, cells were incubated in 250 µM oleate: 2% BSA to lipid load cells. Lipid loading is required to visualize lipid droplets in myotubes, which under lipid-free conditions are sparse. Once loaded cells were then treated for 20 minutes with 2 mM 5-aminoimidazole-4-carboxaminde-1-β-d-ribofuranoside (AICAR), 20 µM forskolin, or 5 mM caffeine. Cells were fixed in 4% PFA before immunohistochemical analyses described above.

### Statistical Analysis

Data are expressed as means ± SEM. Statistical analysis was performed by repeated measures one-way analysis of variance (ANOVA) with a Bonferroni post hoc test, paired or unpaired t-tests where appropriate (GraphPad Prism Version 5.02). Immunohistochemistry images were analyzed for colocalization with ImageJ (NIH). Dual stained images were analyzed with the Image Correlation Analysis plugin utilizing the Manders' colocalization coefficients, M1 and M2. Manders colocalization coefficients, M1 and M2, provide the portion of the signal intensity in one channel that coincides with signal intensity in the other channel assuming there is a difference in the number of objects in both channels [24]. Statistical significance was set *a priori* at P≤0.05.

## Results

### PLIN5 localization in human skeletal muscle at rest and during moderate intensity exercise

**Figure 2:**
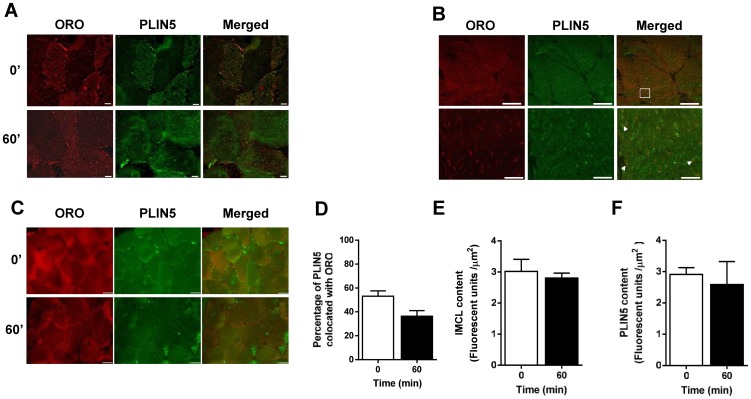
Localization of PLIN5 and lipid droplets in human skeletal muscle sections at rest and immediately after 60 min of moderate intensity exercise. (A) Representative images of one field of view (40× magnification) of human skeletal muscle sections obtained at rest (0′) and after 60 min of exercise of PLIN5 and ORO and merged image (Bar  = 10 µm). (B) Representative images of one field of view (60× magnification, Bar = 50 µm) of human skeletal muscle section with increased magnification below highlighted in white box (Bar  = 10 µm). Arrows highlighting areas of colocalisation. (C) Representative images of one field of view (20× magnification, Bar  = 50 µm) of human skeletal muscle sections obtained at rest (0′) and after 60 min of exercise of PLIN5 and ORO and merged image. (D) The Manders Coefficient (M2) describes the proportion of PLIN5 colocated with ORO. (E) There was no change in intramyocelluar neutral lipid content or (F) PLIN5 content from rest to after exercise. Results shown are means ± SEM derived from 23 optical sections for rest and 20 sections for 60 min, from 6 subjects.

**Figure 3:**
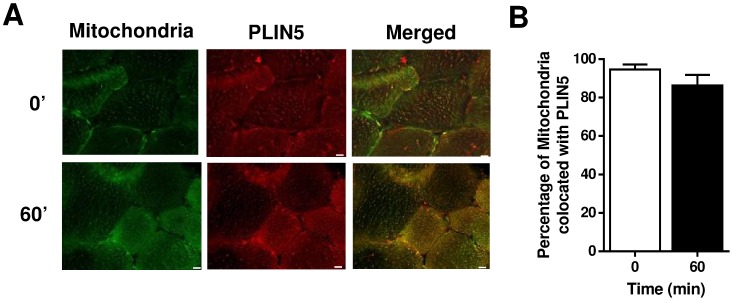
Localization of Mitochondria and PLIN5 in human skeletal muscle sections at rest and immediately after 60 min of moderate intensity exercise. (A) Representative images of one field of view (40× magnification) of human skeletal muscle sections obtained at rest (0′) and after 60 min of exercise (Bar  = 10 µm). (B) The Manders Coefficient (M2) describes the proportion of PLIN5 colocated with mitochondria. Results shown are means ± SEM derived from 16 optical sections for rest and 16 sections for 60 min, from 4 subjects.

We hypothesized that PLIN5 localization and interactions with other lipolytic proteins are important regulators of PLIN5 function in muscle. Skeletal muscle biopsies obtained at rest and during exercise were stained with Oil Red O (ORO) to identify intracellular lipid droplets, and then double labeled to assess protein localization. PLIN5 was expressed uniformly throughout myofibres with increased expression in certain fibres, which were presumably type 1 fibres based on diameter, PLIN5 content and lipid droplet staining [25] (Figure 2A). Lower and higher magnification images showing colocalization are presented in Figure 2B–C. The immunohistochemical analyses in resting tissue shows that 55±4% of PLIN5 colocated with ORO (Figure 2D). There were no changes in PLIN5/ORO colocation during the transition from rest to exercise (0–5 min, not shown) or after prolonged exercise (60 min) (Figure 2D). While intramyocellular triacylglycerol is an important energy source during moderate intensity exercise [26], there was no difference in intramyocellular neutral lipid content between pre- and post-exercise (Figure 2E), presumably due to matched rates of myocellular lipolysis and esterification. Similarly, acute exercise did not alter PLIN5 content (Figure 2F). Although not definitive, this suggests that the relationships reported between lipid droplets and PLIN5 in this study are not impacted by exercise-mediated changes in their contents. PLIN5 associates with the mitochondria and, in this capacity, has been postulated to drive fatty acid oxidation [27]. Colocalization of PLIN5 and mitochondria was not different between rest and exercise (Figure 3A–B). Together, these data suggest that exercise has no effect on PLIN5 localization with lipid droplets or mitochondria.

**Figure 4:**
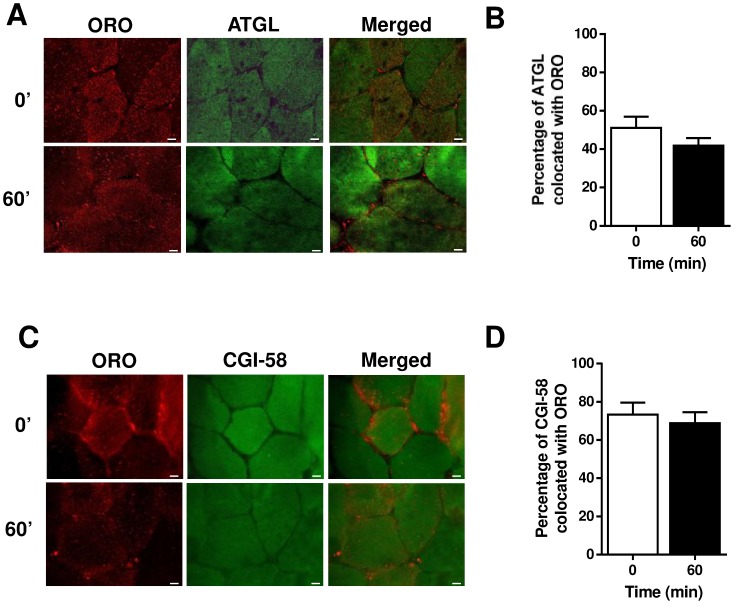
Localization of ATGL and CGI-58 with lipid droplets in human skeletal muscle sections at rest and immediately after 60 min of moderate intensity exercise. (A) Representative images of one field of view (40× magnification) of human skeletal muscle sections obtained at rest (0′) and after 60 min of exercise (Bar  = 10 µm). (B) The Manders Coefficient (M2) describes the proportion of ATGL colocated with ORO. Results shown are means ± SEM derived from 23 optical sections for rest and 20 sections for 60 min, from 6 subjects. Results shown are means ± SEM derived from 24 optical sections all time points, from 6 subjects. (C) Representative images of one field of view (40× magnification) of human skeletal muscle sections obtained at rest (0′) and after 60 min of exercise (Bar  = 10 µm). (D) The Manders Coefficient (M2) describes the proportion of CGI-58 colocated with ORO. Results shown are means ± SEM derived from 24 optical sections all time points, from 6 subjects.

ATGL was localized throughout the myofibre (Figure 4A), with no punctate staining apparent as observed in cultured adipocytes [28]. Approximately 50% of the ATGL colocated with ORO at rest and similar to PLIN5, there were no exercise-mediated changes in ATGL/ORO colocation (Figure 4B). CGI-58 exhibited a similar profile with diffuse staining throughout the myofibre (Figure 4C), with 58±5% colocating with ORO at rest and no changes in colocation with exercise (Figure 4D).

**Figure 5:**
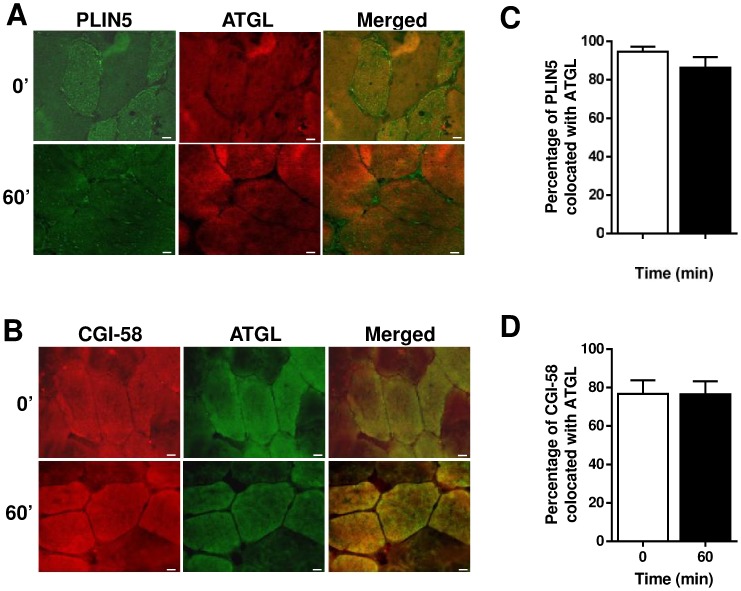
Localization of lipid droplet-associated proteins in human skeletal muscle sections at rest and immediately after 60 min of moderate intensity exercise. Representative images of one field of view (40× magnification) of human skeletal muscle sections obtained at rest (0′) and after 60 min of exercise (Bar  = 10 µm) for (A) PLIN5/ATGL and (B) CGI-58/ATGL. (C) PLIN5/ATGL colocation did not change from rest to exercise as expressed by Manders Colocalization Coefficient, M2. (D) CGI-58/ATGL colocation did not change from rest to exercise as expressed by Manders Colocalization Coefficient, M2. Results for PLIN5/ATGL shown are means ± SEM derived from 19 optical sections at rest and 20 optical sections at 60 min, from 5 subjects. Results for CGI-58/ATGL shown are means ± SEM derived from 8 optical sections at rest and 12 optical sections at 60 min, from 3 subjects.

We next examined colocation of the key lipolytic proteins. The Manders colocalization coefficient was high in all immunohistochemical analyses, supporting the tight relationship between PLIN5, ATGL and CGI-58. PLIN5 exhibited high colocation with ATGL at rest (94±10%) and this was not altered with exercise (Figure 5B). Similarly, ATGL and CGI-58 were highly colocated at rest (76±18%) and, again, this relationship was unchanged during exercise (Figure 5D). We were unable to reproducibly perform dual immunohistochemistry with PLIN5 and CGI-58.

### PLIN5 cellular localization is not altered by pharmacological modulators of fatty acid metabolism in cultured myotubes

**Figure 6:**
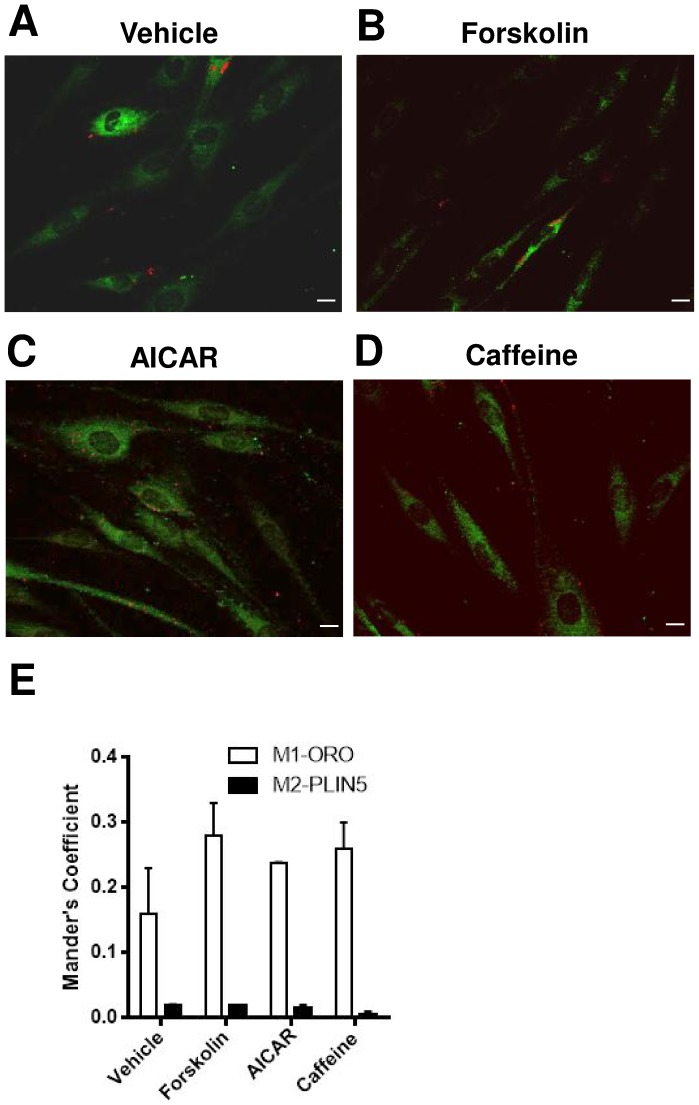
PLIN5 expression in human primary myotubes. (A) Representative merged images of lipid droplets (Red) and PLIN5 (Green) in human primary skeletal muscle myotubes with (A) vehicle, (B) 20 µM forskolin, (C) 2 mM AICAR, and (D) 5 mM caffeine (Scale bar  = 10 µm). (E) PLIN5 and ORO quantified with Manders' coefficient PLIN5 (M1) and ORO (M2).

While our data indicated that PLIN5 localization is not altered during moderate intensity exercise, it was possible that the stress was insufficient to induce detectable changes. To determine whether cellular localization of PLIN5 is altered under conditions of stimulation or inhibition of lipolytic/fatty acid oxidation pathways, human primary myocytes were acutely treated with 2 mM AICAR (5′-AMPK activator that increases fatty acid oxidation), 20 µM forskolin (protein kinase A activator that increases lipolysis), and 5 mM caffeine (CaMK activator). Approximately 20% of ORO colocated with PLIN5, while less than 2% of PLIN5 colocated with ORO (Figure 6), indicating that the vast majority of PLIN5 is not lipid droplet associated in primary human myocytes. Colocalization of PLIN5 with the stained lipid droplet did not differ between untreated and stimulated conditions. The difference between the proportions of ORO/PLIN5 colocation could be attributed to the lower amount of lipid droplets and TG found in the human primary myotubes, despite prior lipid loading.

## Discussion

Lipolysis is a highly conserved process that is essential for the supply of fatty acid substrate both at rest and during times of increased physiological demand such as fasting, cold exposure and prolonged exercise. The oxidation of fatty acids released from lipolysis of intramyocellular triacylglycerol can contribute up to 55% of the total energy expenditure during moderate intensity exercise [26], [29], [30], making it an important metabolic substrate. Dysregulated intramyocellular triacylglycerol lipolysis is associated with the development of insulin resistance [31], [32] which has precipitated interest in understanding lipid droplet fatty acid fluxes in the context of diabetes development [33]. The regulation of muscle lipolysis remains incompletely defined, owing in part to a paucity of information regarding the interactions between key lipolytic effectors. The colocation studies in human skeletal muscle reported here indicate that there is an abundance of the key lipolytic proteins ATGL, PLIN5 and CGI-58 that colocate with triacylglycerol and that these lipolytic modulators are likely to be physically associated at rest. Unexpectedly, there was no evidence of increased localization of the lipolytic proteins with triacylglycerol during exercise, nor was there an increased colocation of ATGL with its coactivator CGI-58, or ATGL with PLIN5.

Renewed interest in the field of muscle lipolysis has been stimulated by the discovery of PLIN5, an intracellular protein that is highly expressed in muscle that shares close homology with the major regulatory protein of adipocyte lipolysis, PLIN1 [34]. The regulation and role of PLIN5 is unresolved and controversial. Studies conducted in a variety of immortalized cell lines, isolated muscle and using forced expression of PLIN5 and other lipolytic regulators demonstrate that PLIN5 interacts with ATGL and CGI-58 independently and concentrates them at lipid droplets to enhance ATGL activity and lipolysis [16], [20], [35], [36]. Others using similar approaches have shown that PLIN5 recruits ATGL to lipid droplets and plays a negative role in lipolysis by inhibiting ATGL activity; whereas CGI-58 recruits ATGL to the lipid droplet and increases lipolysis [37]. To add complexity, another study suggests that PLIN5 resides in high-density lipid droplets and promotes lipid storage when fatty acids are in excess [13]. While seemingly disparate, these latter studies may explain the initial perplexing finding that PLIN5 can both increase fatty-acid induced triacylglycerol storage and fatty acid oxidation [11] and in this way maintain intracellular fatty acids below ‘lipotoxic’ levels. Our data show that both PLIN5 and ATGL, and CGI-58 and ATGL are colocated with lipid droplets (ORO) at rest. The abundance of lipolytic regulators located at the lipid droplet would agree with the high turnover rate of triacylglycerol in skeletal muscle [38]. We cannot determine ATGL, PLIN5 and CGI-58 colocation due to technical constraints nor can we determine the functional relevance of these associations in humans *in vivo*. Our data also show that the colocation of PLIN5 with lipid droplets is not altered during moderate intensity exercise, which agrees with previous reports in isolated contracting rat muscle [19] and suggests that the amount of PLIN5 at the lipid droplet does not change during increased lipolytic flux. The PLIN5/ATGL colocation was similarly unaffected during exercise, which leads to the presumptuous interpretation, that PLIN5 does not inhibit ATGL action. However, such a conclusion is premature given that other regulatory factors are likely to modulate PLIN5/ATGL outcomes, such as PKA-mediated phosphorylation of PLIN5 which increases ATGL mediated lipolysis [37] and ATGL phosphorylation [39] which may alter the PLIN5/ATGL interaction.

PLIN5 is postulated to increase fatty acid oxidation by facilitating the transfer of fatty acids from lipid droplets to the mitochondria [40]. This is based on several complimentary observations: PLIN5 is highly expressed in oxidative and not glycolytic tissues [9]–[11]; lipid droplets and the mitochondria are spatially associated in muscle and PLIN5 is located near both organelles [14]; PLIN5 may recruit mitochondria to lipid droplets [40]; and overexpression of PLIN5 may promote an oxidative phenotype [27], although the latter point is not supported by several other studies [14], [41], [42]. We reasoned that acute moderate intensity exercise, which increases fatty acid oxidation rates by ∼5-10-fold (calculated from [43]), would provide an ideal platform to test this hypothesis. Our data show that the PLIN5/mitochondria colocation is not different between resting and exercise conditions, indicating that PLIN5 is unlikely to be mediating marked changes in lipid droplet-mitochondria flux due to increased abundance at the mitochondria. This conclusion is only applicable in the context of acute exercise in humans.

ATGL is an important regulator of triacylglycerol lipolysis in all tissues, including skeletal muscle [44]–[46], and studies in adipocytes indicate that ATGL translocates from a cytosolic location to the surface of lipid droplets during PKA-stimulated lipolysis [28], [47], [48]. As discussed above, the translocation of ATGL permits interaction with CGI-58 and increases lipase activity [16], [49]. We are unaware of any previous study that has examined ATGL localization in skeletal muscle. Our studies indicate that ATGL is localized diffusely throughout the myofibre, with no evidence of ‘punctate’ staining around ORO stained lipid droplets. While this does not agree with the clear evidence in cultured adipocytes demonstrating that ATGL and CGI-58 associates to increase ATGL activity upon β-adrenergic stimulation, significant differences exist between studies. Firstly, adipocytes store triglyceride substrate in a prominent, single lipid droplet that accounts for >90% of cellular mass, whereas lipid droplets are scattered throughout myofibres. Hence, this morphological difference can explain the diffuse localization of lipid droplet proteins throughout myofibres compared with adipocytes. Secondly, cell-based studies typically use pan-β-adrenergic agonists at low µM (pharmacological) concentrations to activate PKA signaling and lipolysis, whereas the circulating catecholamine levels during moderate exercise is low nM. Thirdly, ATGL (and PLIN5, CGI-58) have functions other than lipolytic regulation, which supports the premise that the proteins should not be expected to be localized to one cellular location. Finally, we question the relevance of the cultured adipocyte system when examining muscle lipolysis and are, in fact, unaware of evidence supporting marked ATGL translocation to the lipid droplet in adipocytes upon physiological PKA activation *in vivo*. In this study, we also show that ATGL colocation with ORO and CGI-58 do not change during exercise, suggesting that sufficient protein is present at its substrate to modulate lipolytic rates. A caveat to these interpretations is that the immunohistochemistry approaches we employed permit conclusions of colocation and not direct physical interaction. Nevertheless, the complete absence of change in colocation between ATGL/ORO and ATGL/CGI-58 between rest and exercise support our tentative conclusions. By contrast, ATGL and CGI-58 were shown to associate during contraction-induced lipolysis in isolated rat muscle [20]. The exercise modality (moderate whole-body exercise vs. heavy electrical stimulation), species (human vs. rat) or analytical methods (immunohistochemistry vs. immunoblotting) could contribute to the differences between these studies. A technical limitation of the immunohistochemistry approach is that very small lipid droplets are not visible using ORO staining; hence, it is possible that some protein/ORO colocalization may be underestimated in the present study.

An unrelated finding of interest was that only 50% of the ATGL associated with ORO in skeletal muscle, suggesting that discrete pools of ATGL may perform distinct cell functions. ATGL possesses transacylase activity [50] and may act as a receptor for the protein pigment epithelium-derived factor [51] to enhance lipolysis. This could explain both ER and plasma membrane localization of ATGL in muscle. Further studies are required to address the putative involvement of ATGL in non-lipase related cell functions.

The interaction between PLIN5, ATGL and CGI-58 are complex, as is the role of PLIN5 is regulating triacylglycerol metabolism. Cell-based studies have begun to unravel some of these complexities and our studies in humans have shed new light on the *in vivo* relevance of these relationships in skeletal muscle [22]. The major lipolytic proteins are highly expressed at the lipid droplet, coassociate in resting skeletal muscle and their localization and interactions appear to remain unchanged during prolonged exercise. We speculate that there is sufficient ‘machinery’ localized to lipid droplets to maintain adequate lipolytic flux in skeletal muscle at rest and other post-translational mechanisms may regulate the increased lipolytic flux during exercise. In this context, ATGL [22], [39], PLIN5 [36], [37] and CGI-58 (unpublished observations) are phosphorylated by PKA (and possibly other kinases) and future studies are required to elucidate how phosphorylation modulates their activities and interactions in muscle.
